# Evidence for the involvement of NADPH oxidase in adenosine receptor-mediated control of coronary flow using A_1_ and A_3_ knockout mice

**DOI:** 10.1002/phy2.70

**Published:** 2013-08-29

**Authors:** Mohammed S El-Awady, Uthra Rajamani, Bunyen Teng, Stephen L Tilley, S Jamal Mustafa

**Affiliations:** 1 Department of Physiology and Pharmacology, Center for Cardiovascular and Respiratory Sciences and Clinical & Translational Science Institute, West Virginia UniversityMorgantown, West Virginia, 26505; 2 Department of Pharmacology and Toxicology, Faculty of Pharmacy, Mansoura UniversityMansoura, 35516, Egypt; 3 Department of Medicine, University of North CarolinaChapel Hill, North Carolina, 27599

**Keywords:** Adenosine, coronary artery, isolated heart, knockout mice, NADPH oxidase

## Abstract

The NADPH oxidase (Nox) subunits 1, 2 (gp91 *phox*), and 4 are the major sources for reactive oxygen species (ROS) in cardiovascular system. In conditions such as ischemia–reperfusion injury, and hypoxia, both ROS and adenosine are released suggesting a possible interaction. We hypothesized that ROS generated through Nox is involved in adenosine-induced coronary flow (CF) responses. Adenosine (10^−8^–10^−5.5^ mol/L) increased CF in isolated hearts from wild-type (WT; C57BL/6), A_1_ adenosine receptor (AR) knockout (A_1_KO), A_3_AR KO (A_3_KO) and A_1_ and A_3_AR double KO (A_1_/A_3_DKO) mice. The Nox inhibitors apocynin (10^−5^ mol/L) and gp91 ds-tat (10^−6^ mol/L) or the superoxide dismutase (SOD) and catalase-mimicking agent EUK134 (50 μmol/L) decreased the adenosine-enhanced CF in the WT and all the KOs. Additionally, adenosine increased phosphorylation of p47-phox subunit and extracellular signal-regulated kinase (ERK) 1/2 without changing protein expression of Nox isoforms in WT. Moreover, intracellular superoxide production was increased by adenosine and CGS-21680 (a selective A_2A_ agonist), but not BAY 60-6583 (a selective A_2B_ agonist), in mouse coronary artery smooth muscle cells (CASMCs) and endothelial cells (CAECs). This superoxide increase was inhibited by the gp91 ds-tat and ERK 1/2 inhibitor (PD98059). In conclusion, adenosine-induced increase in CF in isolated heart involves Nox2-generated superoxide, possibly through ERK 1/2 phosphorylation with subsequent p47-phox subunit phosphorylation. This adenosine/Nox/ROS interaction occurs in both CASMCs and CAECs, and involves neither A_1_ nor A_3_ ARs, but possibly A_2A_ ARs in mouse.

## Introduction

Adenosine is an autacoid that plays an important role in the control of coronary flow (CF) under different metabolic conditions (Berne 1963). The effects of adenosine are mediated by activation of four well-known cell surface receptors (A_1_, A_2A_, A_2B_, A_3_) (Tabrizchi and Bedi 2001; Mustafa et al. 2009). The role of adenosine receptors (ARs) in control of CF has been studied in several species, with A_2A_ and A_2B_ ARs increasing CF (Flood and Headrick 2001; Sharifi Sanjani et al. 2011; Sharifi-Sanjani et al. 2013) while A_1_ and A_3_ARs decrease CF (Talukder et al. 2002; Sato et al. 2005; Tawfik et al. 2006).

ARs-induced coronary responses are mediated through several effector pathways, such as cyclic adenosine 5′-monophosphate (cAMP) and potassium (K^+^) channels (Fredholm et al. 2000). Reactive oxygen species (ROS) may also play an important role in adenosine signaling pathways. In conditions such as ischemia–reperfusion and hypoxia, both ROS and adenosine are released suggesting a possible interaction between them (Zatta and Headrick 2005; Gebremedhin et al. 2010).

In the vasculature, NADPH oxidases (Nox) are the major source of ROS that play both physiological and pathophysiological roles in the control of vascular tone (Carlstrom et al. 2009). The family of Noxs consists of seven members, Nox1–Nox5, Doux1, and Doux2, among which Nox1, 2 (gp91 *phox*), and 4 are of relevance in the cardiovascular system (Schroder 2010). Several studies have shown that adenosine affects ROS generation through the regulation of Nox activity. Inhibition or knockout of Nox leads to prevention of adenosine responses in aorta (El-Awady et al. 2011), renal arterioles (Carlstrom et al. 2009), and in cerebral arteries (Gebremedhin et al. 2010), confirming an important link between ROS and adenosine.

Although ROS have been linked to adenosine responses in different tissues, little is known about the involvement of ROS in adenosine-induced CF responses. A_2A_ARs, through MAPK signaling, were shown to regulate mouse cardiac ROS production by Nox (Ribe et al. 2008) and to attenuate reperfusion injury partly by inhibiting superoxide generation (Jordan et al. 1997). A_2B_ARs were shown to inhibit superoxide production from mitochondrial complex I in rabbit cardiomyocytes (Yang et al. 2011). A_1_AR activation have been shown to reduce ROS and attenuates stunning in rat ventricular myocytes (Narayan et al. 2001), while inhibiting hydrogen peroxide-induced stimulation of L-type calcium current in guinea pig ventricular myocytes (Thomas et al. 1998).

Recently from our laboratory, A_3_ARs activation was shown to induce contraction of the mouse aorta that is dependent on ROS generation, possibly through Nox2 (El-Awady et al. 2011). We hypothesized that ROS generation through Nox is involved in adenosine-induced CF responses. As both A_1_ and A_3_ ARs have been shown to have signaling through ROS (Narayan et al. 2001; El-Awady et al. 2011), therefore we examined the contribution of these inhibitory ARs (A_1_ and A_3_) on adenosine-induced ROS generation and vasodilation in this study. Additionally, the signaling mechanisms for both A_1_ and A_3_ ARs in relation to ROS are not clear. We used isolated hearts from wild-type (WT), A_1_AR (A_1_KO), and A_3_AR (A_3_KO) knockouts and A_1_ and A_3_AR double knockout (A_1_/A_3_DKO) mice for these studies.

Our data indicate that ROS generated through Nox, mainly Nox2, play an important role in adenosine-induced coronary vasodilation. This effect is not mediated through neither A_1_ nor A_3_ARs, but possibly through A_2A_ AR activation.

## Material and Methods

### Materials

DHE was purchased from Invitrogen (Carlsbad, CA), gp91 ds-tat from Anaspec (Fremont, CA), and EUK134 from Cayman Chemical (Ann Arbor, MI). BAY 60-6583 was a gift from Bayer AG (Leverkusen, Germany). All other chemicals were purchased from Sigma-Aldrich (St. Louis, MO). Stock solutions of adenosine, apocynin, BAY 60-6583, CGS-21680, PD98059 and EUK134 were made in dimethyl sulfoxide (DMSO), whereas gp91 ds-tat was dissolved in distilled water.

### Animals

All of the animal care and experimental protocols were performed according to the guidelines and approval of the Animal Care and Use Committee at West Virginia University. WT (C57BL/6) mice were purchased from The Jackson Laboratory (Bar Harbor, ME). A_1_KO, A_3_KO, and A_1_/A_3_DKO mice of the same background (backcrossed 12 generations to the C57 BL/6 background) were generously provided by Dr. S. Tilley.

### Langendorff-perfused mouse heart preparation

Mice were anesthetized with pentobarbital sodium (50 mg/kg i.p.). Hearts were rapidly removed into heparinized (5 U/mL) ice-cold Krebs–Hensleit (KH) buffer containing (in mmol/L) 119 NaCl, 11 glucose, 22 NaHCO_3_, 4.7 KCl, 1.2 KH_2_PO_4_, 1.2 MgSO_4_, 2.5 CaCl_2_, 2 pyruvate and 0.5 EDTA. Hearts were retrogradely perfused through the aorta cannulated with a 20-gauge, blunt-ended needle at a constant pressure of 80 mmHg and continuously gassed with 95% O_2_–5% CO_2_ KH buffer at 37°C. The left atrium was removed, and a water-filled balloon was inserted into the left ventricle across the mitral valve and connected to a pressure transducer permitting continuous measurement of left ventricular developed pressure (LVDP). Balloon volume was modified to maintain a left ventricular diastolic pressure of 2–5 mmHg. CF was measured via a Transonic flow probe (Transonic Systems, Ithaca, NY) in the aortic perfusion line. Baseline CF, LVDP, and heart rate (HR) were monitored continuously and recorded on a Power Lab data-acquisition system (AD Instruments, Colorado Springs, CO). Hearts were allowed to equilibrate for 30 min before starting experimental protocols.

### Experimental protocol

After baseline data were acquired, concentration-response curve (CRC) was performed by exposing each heart to progressively increasing concentrations of adenosine (10^−8^–10^−5.5^ mol/L). Each concentration of adenosine was infused for 5 min, followed by a minimum of 5 min of perfusion for drug washout. In separate experiments, the Nox inhibitor apocynin (10^−5^ mol/L), the specific Nox-2 inhibitor gp91 ds-tat (10^−6^ mol/L) or the superoxide dismutase (SOD), and catalase-mimicking drug EUK134 (50 μmol/L) were perfused for 20 min before and during adenosine CRC. All compounds were infused at a rate of 1/100 of the CF through an injection port directly proximal to the aortic cannula using a Genie Plus Syringe pump (Kent Scientific, Torrington, CT).

### Immunoblotting

After each experiment, hearts were rapidly collected, snap frozen in liquid nitrogen and kept at −80°C. Hearts were homogenized with 10 volumes of ice-cold RIPA buffer (Cell Signaling, Danvers, MA) with 1% Halt Protease inhibitor cocktail (Thermo Scientific, Rockford, IL) and 1 mmol/L sodium fluoride. Homogenized samples were centrifuged for 30 min at 12,000 *g* at 4°C. The protein content of the supernatant was determined using Bradford protein assay (BioRad, Hercules, CA).

Aliquots of the heart lysates (30 μg protein/well) were separated on NuPAGE 4–12% bis-Tris Gels (Invitrogen, Grand Island, NY). Prestained Novex Sharp Protein Standards (3.5–260 kDa, Invitrogen) were run in parallel. Proteins were transferred to nitrocellulose membranes then incubated with 5% milk for 1 h to block nonspecific binding sites. Membranes were then probed with either anti-gp91 phox (anti-Nox2) mouse monoclonal IgG (BD Biosciences, San Jose, CA), anti-Nox4 rabbit polyclonal IgG (Abcam, Cambridge, MA), anti-p47-phox rabbit polyclonal IgG (Santa Cruz Biotechnology, Santa Cruz, CA), anti-phospho extracellular signal-regulated kinase 1/2 (ERK 1/2) mouse monoclonal IgG (Santa Cruz Biotechnology) or anti-ERK 1/2 rabbit polyclonal IgG (Santa Cruz Biotechnology) at a dilution of 1:1000; anti-Nox1 rabbit polyclonal IgG (Abcam) at a dilution of 1:500, followed by incubation with secondary antibodies (horseradish peroxidase-conjugated goat anti-mouse or goat anti-rabbit; Santa Cruz Biotechnology) at 1:10,000 dilution for 1 h. After extensive washing, membranes were then stripped and reprobed with monoclonal anti-β-actin antibody (Santa Cruz Biotechnology) at a dilution of 1:20,000. For detection of bands, the membranes were treated with enhanced chemiluminescence plus (for Nox2, Nox4, p47-phox, phospho ERK 1/2, ERK 1/2, and β-actin) or advance (for Nox1) kits (GE Healthcare, Buckinghamshire, U.K.) for 1 min and subsequently exposed to ECL Hyperfilm. Relative band intensities were quantified by densitometric analysis (ImageJ 1.43u, NIH), and each sample was normalized as a ratio to either the β-actin, total p47-phox or total ERK 1/2 values as appropriate.

### Isolation of phosphoproteins

Phosphoproteins were isolated from some hearts after the experiments using phosphoprotein purification kit (Qiagen, Hilden, Germany). Total and phospho proteins were separated on the same gel, transferred, and probed with anti-p47-phox rabbit polyclonal IgG (Santa Cruz Biotechnology) as previously mentioned in immunoblotting.

### Measurement of superoxide generation in mouse coronary artery

Mouse coronary arteries were isolated from WT immediately prior to the experiment. The left and the right coronary branches were employed as no differences were found between left and right branches. The coronary arteries were put in culture media (Dulbecco's modified Eagle's medium + 10% fetal bovine serum) (ATCC, Manassas, VA) then treated with 25 μmol/L dihydroethidium (DHE) and incubated for 40 min. The arteries were then pinned and secured on a petri dish and then washed for 10 min with phosphate buffer solution. Thereafter, the arteries were maintained and treated in the same culture media until the end of the experiment. The arteries were viewed using Zeiss Violet Confocal microscope (LSM510; Heidelberg, Germany) at 40× magnification using a dipping lens (Ex/Em 480/590). An initial image of the arteries was taken under the control condition. Each artery served as its own control and treatment -related changes in ROS were compared to its own control. Once the control image was acquired, the arteries were subject to various treatments. They were treated with adenosine (10^−5^ mol/L), CGS-21680 (10^−6^ mol/L, A_2A_ selective agonist) or BAY 60-6583 (10^−6^ mol/L, A_2B_ selective agonist) for 10 min and images obtained. In separate sets of experiments, the arteries were also treated with Nox inhibitor gp91 ds-tat (10^−6^ mol/L) or ERK 1/2 inhibitor (PD98059, 10^−5^ mol/L) for 20 min and images obtained before the addition of adenosine, CGS-21680 or BAY 60-6583. Hydrogen peroxide (200 μmol/L) served as the positive control (∼40% increase in intensity). To correct for the effects of quenching, a timeline control was done for 40 min to note the percentage changes in fluorescence every 5 min during the course of the experiment. On each segment of the artery, smooth muscle cells (found across the artery) and endothelial cells (found along the artery) were individually chosen and fluorescence intensity was obtained. On each treatment condition per artery the same cells were assessed for differences in ROS levels. An “*n*” of at least four animals was employed. Mean fluorescence intensity was calculated using ImageJ software.

### Statistical analysis

Data were expressed as mean ± standard error of mean (SEM), where (*n*) equals the number of animals. The highest CF response obtained in the Langendorff experiments was considered as the maximum response (E_max_). Statistical analysis was carried out using Graphpad Prism software (Graphpad Software Inc., San Diego, CA). Significant differences between groups were determined with unpaired Student's *t*-test or one-way analyses of variance (ANOVA) with Tukey-Kramer's Multiple Comparisons post hoc test as appropriate. Significance level was considered when *P* < 0.05.

## Results

### Baseline functions of isolated hearts of WT, A_1_KO, A_3_KO, and A_1_/A_3_DKO mice

Significant baseline CF differences (*P* < 0.05, *n* = 6) were observed in WT, A_1_KO, and A_1_A_3_DKO. A_1_KO and A_1_A_3_DKO had a significantly increased baseline coronary flow compared to WT animals. No significant differences were found in HR, LVDP, animal weights or heart weights between any of the KO and the WT hearts (Table 1).

**Table 1:** Baseline data for WT, A_1_KO, A_3_KO, and A_1_/A_3_DKO mice isolated hearts (Langendorff)

	WT	A_1_KO	A_3_KO	A_1_/A_3_DKO
Age, weeks	15.3 ± 0.14	15.8 ± 0.4	15.6 ± 0.2	16.2 ± 0.3
No. of mice	30	18	18	18
Body weight, g	22.9 ± 1.0	22.7 ± 1.3	21.6 ± 0.9	22.8 ± 1.1
Heart weight, mg	99.2 ± 1.9	99.8 ± 2.6	100.1 ± 3.6	98.9 ± 3.1
Heart weight-to-body weight ratio,%	0.43 ± 0.02	0.427 ± 0.01	0.437 ± 0.013	0.419 ± 0.01
Coronary flow, mL/min/g heart weight	13.54 ± 1.02	19.56 ± 1.72*	16.53 ± 2.18	17.26 ± 1.26*
Heart rate, beats/min	349 ± 12	374 ± 7	363 ± 13	400 ± 7
Left ventricular developed pressure, mmHg	98 ± 6	95 ± 5	102 ± 6	105 ± 7

Values are mean ± SEM, *n* = 6. All parameters were collected after 30 min of equilibration. WT (C57BL/6); A_1_KO (A_1_ AR knockout); A_3_KO (A_3_AR knockout) and A_1_/A_3_DKO (A_1_ and A_3_AR double knockout mice).

* A1KO and A1A3DKO had significantly higher baseline flow compared to WT.

### Effect of different Nox inhibitors on adenosine-mediated CF responses in WT mice isolated hearts

**Figure 1:**
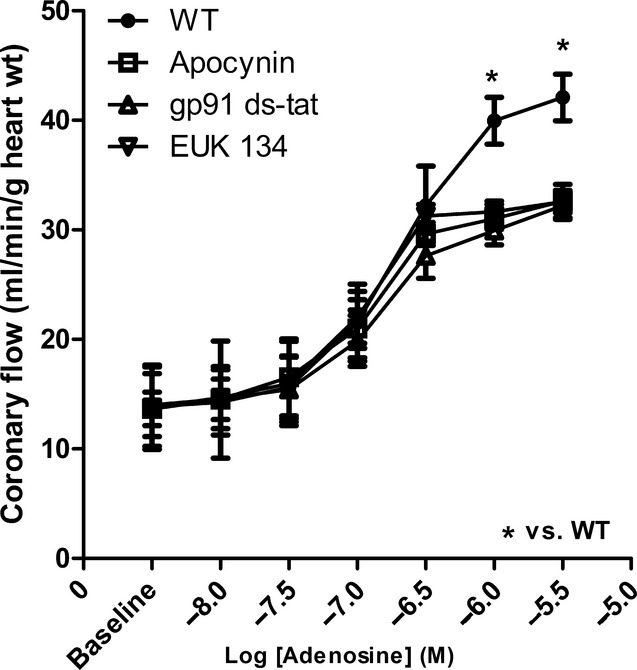
Effects of the Nox inhibitors, apocynin (10^−5^ mol/L), gp91 ds-tat (10^−6^ mol/L) and the SOD and catalase-mimicking agent EUK134 (50 μmol/L) on CF changes induced by adenosine in WT hearts. Data are expressed as mean ± SEM (*n* = 6). *P < 0.05.

Adenosine caused a concentration-dependent increase in CF in WT mice (Fig. 1), with a maximum increase in CF by 270% from the baseline (100%). Inhibition of Nox by apocynin (10^−5^ mol/L) or gp91 ds-tat (10^−6^ mol/L) significantly (*P* < 0.05, *n* = 6) decreased the enhanced CF to adenosine, where the E_max_ dropped from 270% to 220% (Fig. 1). This suggests that Nox activation is involved in adenosine-mediated CF responses. In addition, the SOD and catalase-mimicking agent, EUK134 had effects similar to Nox inhibitors on adenosine-induced increase in CF in WT hearts (Fig. 1). EUK134 decreased the E_max_ for adenosine CRC to 213% from the baseline, confirming that ROS, probably produced from Nox, are involved in adenosine-induced increase in CF in WT hearts. All of these inhibitors had no effect on adenosine-induced changes in either the HR or the LVDP.

### Effect of the Nox inhibitors on adenosine-mediated CF responses in A_1_KO, A_3_KO, and A_1_/A_3_DKO mice isolated hearts

**Figure 2:**
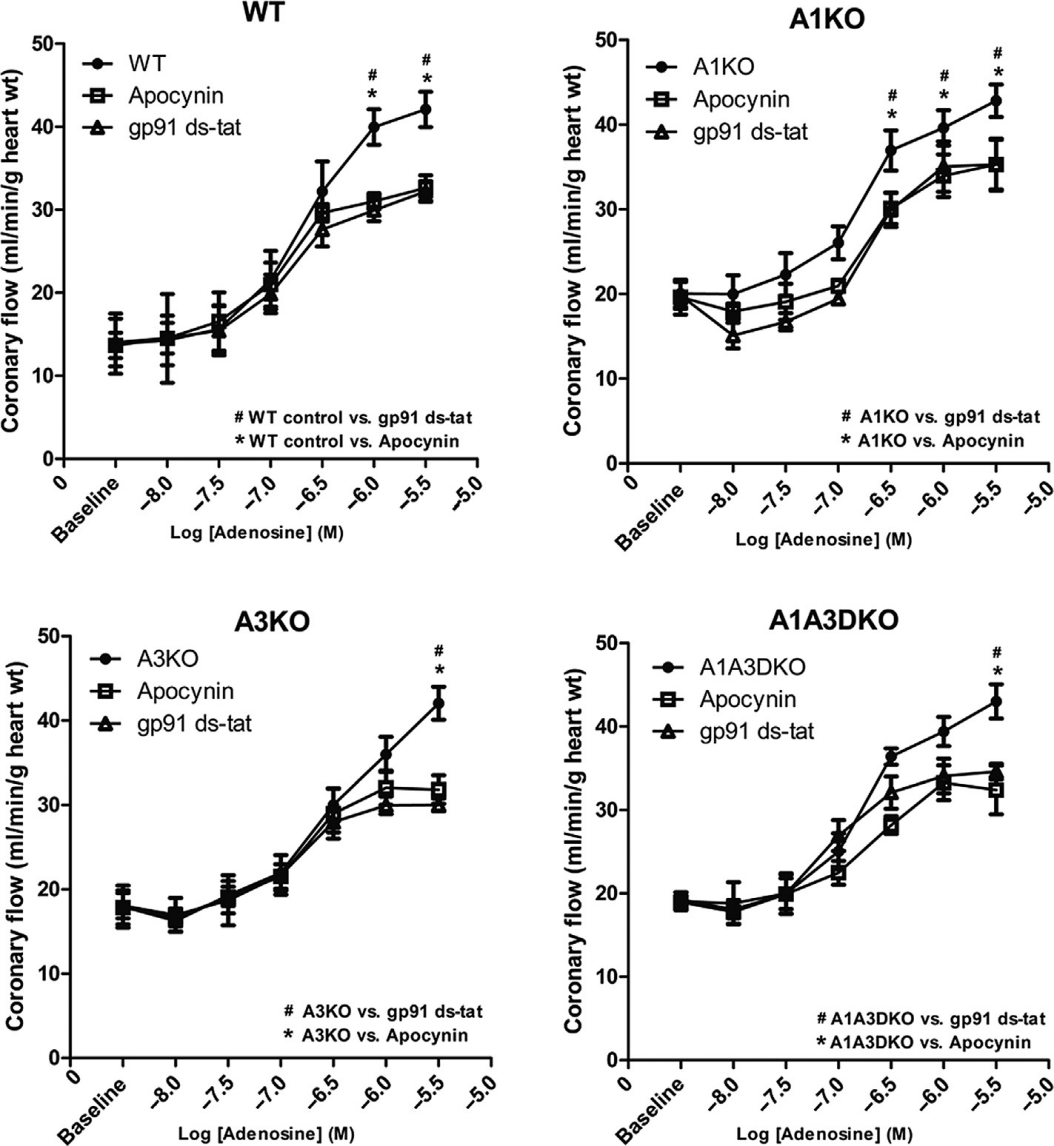
Effect of the Nox inhibitor apocynin (10^−5^ mol/L) and gp91 ds-tat (10^−6^ mol/L) on CF changes induced by adenosine in WT, A_1_KO, A_3_KO, and A_1_/A_3_DKO hearts. Data are expressed as mean ± SEM (*n* = 6). *,# *P* < 0.05.

Similar to WT isolated hearts, adenosine caused a concentration-dependent increase in CF (Fig. 2), with the maximum increase of 221, 241, and 233% from the baseline in A_1_KO, A_3_KO, and A_1_/A_3_DKO isolated hearts, respectively. Inhibition of Nox by apocynin (10^−5^ mol/L) or gp91 ds-tat (10^−6^ mol/L) significantly (*P* < 0.05, *n* = 6) decreased the adenosine-mediated increase in CF (Fig. 2). The E_max_ for adenosine was reduced by apocynin to 178, 194, and 188% and by gp91ds-tat to 178, 176, and 204% from the baseline in A_1_KO, A_3_KO and A_1_/A_3_DKO isolated hearts, respectively. This suggests that neither A_1_ nor A_3_AR is involved in adenosine-mediated increase in CF. Adenosine-induced changes in HR and LVDP were not affected by apocynin or gp91 ds-tat (10^−6^ mol/L) in A_1_KO, A_3_KO, and A_1_/A_3_DKO isolated hearts.

### Effect of adenosine on protein expression of Nox isoforms and phosphorylation of p47-phox in WT isolated hearts

**Figure 3:**
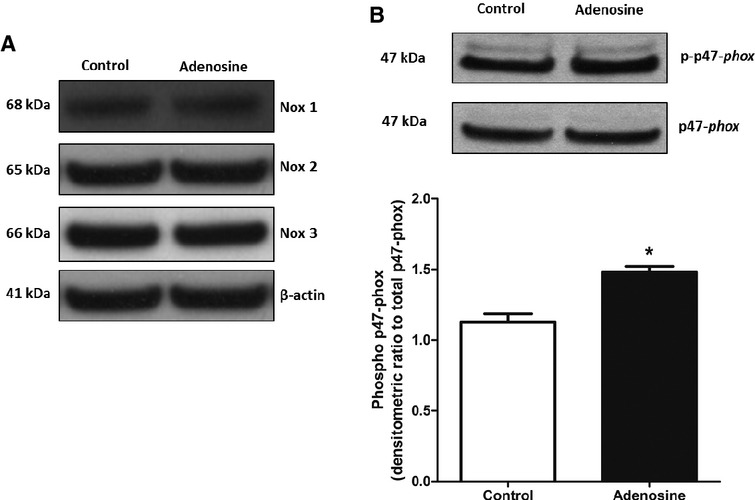
(A) Representative blots for changes in protein expression for Nox1, 2, and 4 induced by adenosine in WT hearts. (B) Representative blots and densitometric analysis for changes in phosphorylation of p47-phox induced by adenosine in WT hearts. Data are expressed as mean ± SEM (*n* = 4). *P < 0.05, significantly different compared to control using unpaired *t*-test.

For further confirmation of the role of Nox in CF increase and ROS generation induced by adenosine, the protein expression levels of Nox1, 2, and 4 subunits were examined from isolated hearts. Nox1, 2, and 4 protein expression was not affected by adenosine (10^−5^ mol/L) treatment in WT isolated hearts (Fig. 3A). However, adenosine significantly (*P* < 0.05, *n* = 4) increased the phosphorylation of the Nox subunit p47-phox by 20% in WT mice (Fig. 3B). These data suggest that adenosine treatment may enhance Nox activation through increasing p47-phox phosphorylation but not through changes in protein expression of Nox isoforms.

### Effect of adenosine on phosphorylation of ERK 1/2 in WT isolated hearts

**Figure 4:**
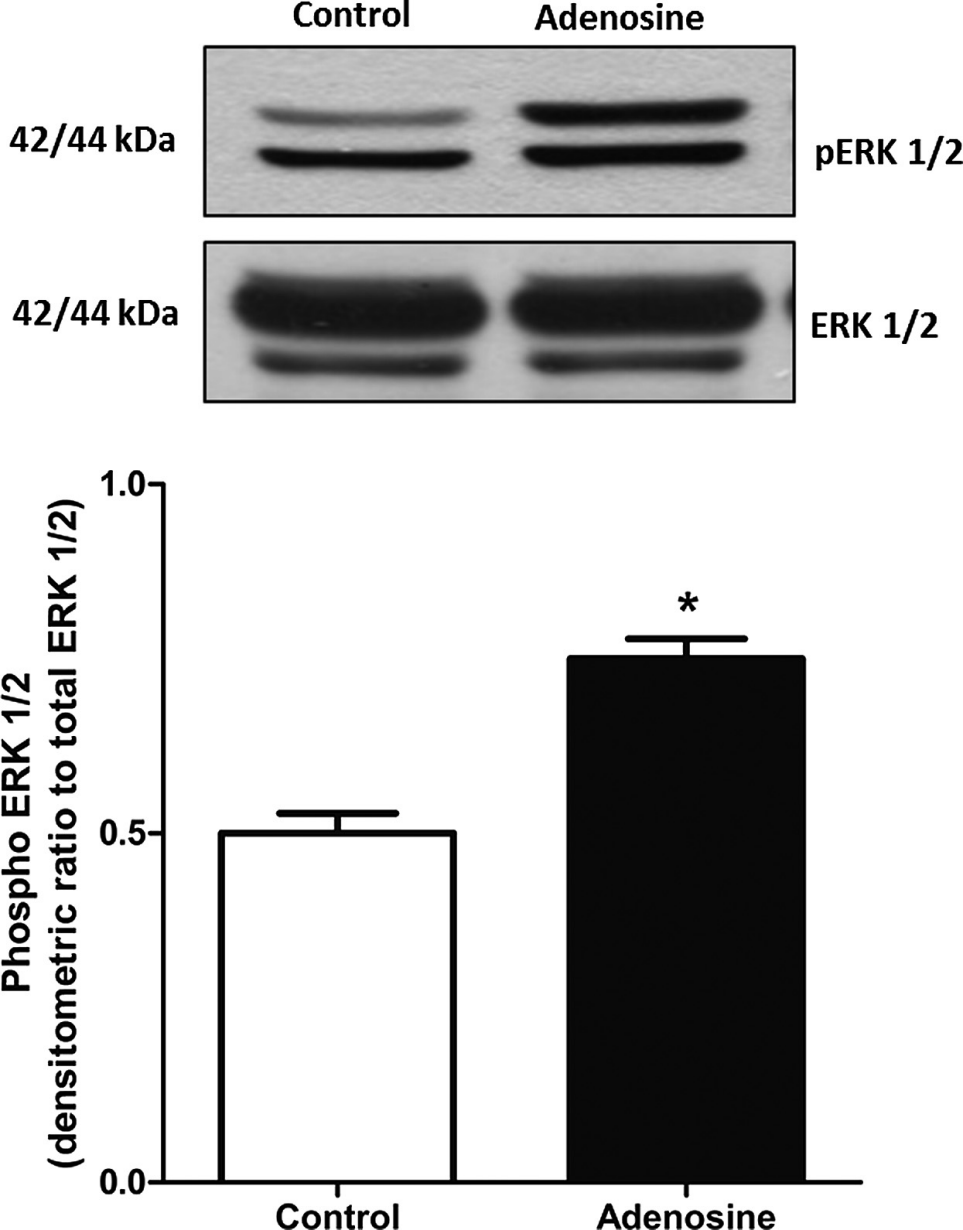
Representative blots and densitometric analysis for changes in phosphorylation of ERK 1/2 induced by adenosine in WT hearts. Data are expressed as mean ± SEM (*n* = 4). *P < 0.05, significantly different compared to control using unpaired *t*-test.

ERK 1/2 phosphorylation was examined in isolated hearts as a possible signaling pathway for ROS. Adenosine significantly (*P* < 0.05, *n* = 4) increased the phosphorylation of the ERK 1/2 by 67% in WT mice (Fig. 4). These data suggest that ERK 1/2 phosphorylation may be a possible signaling pathway for ROS and adenosine in isolated heart. This is further supported by the data presented below.

### Effect of the Nox and ERK 1/2 inhibitors on superoxide generation by adenosine and CGS-21680 from mouse coronary artery

**Figure 5:**
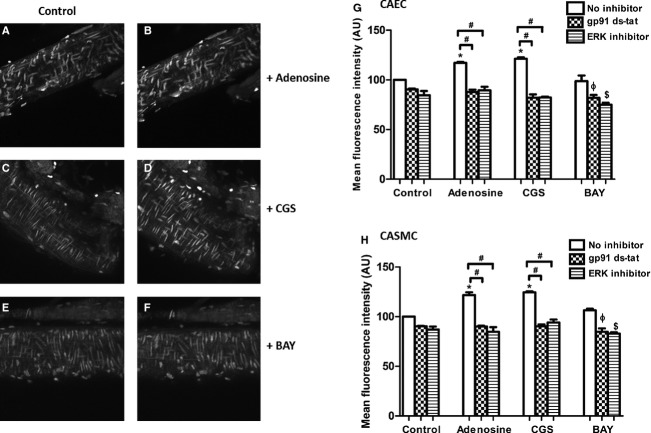
Effects of the Nox inhibitor gp91 ds-tat (10^−6^ mol/L) and the ERK 1/2 inhibitor PD98059 (10^−5^ mol/L) on intracellular superoxide production. (A), (C), and (E) are the untreated controls. (B) (adenosine; 10^−5^ mol/L), (D) (CGS-21680; 10^−6^ mol/L, CGS), and (F) (BAY 60-6583; 10^−6^ mol/L) are their respective treatments in mouse coronary arteries. Data are expressed as mean ± SEM (*n* = 4). (G) Coronary Artery Endothelial Cells (CAEC): * vs. Control – no inhibitor, ϕ vs. Adenosine + gp91 ds-tat, $ vs. Control + ERK inhibitor; (H) Coronary Artery Smooth Muscle Cells (CASMC): * vs. Control – no inhibitor, ϕ vs. Adenosine + gp91 ds-tat, $ vs. Control + ERK inhibitor

Adenosine (10^−5^ mol/L) treatment increased superoxide levels by 20% in coronary smooth muscle cells and 16% in coronary endothelial cells (*P* < 0.05, *n* = 4) versus untreated controls (Fig. 5B, G and H). The selective A_2A_ agonist CGS-21680 (10^−6^ mol/L) treatment showed a 24% increase in superoxide levels in coronary smooth muscle cells and a 20.75% increase in endothelial cells compared to untreated control (Fig. 5D, G and H). The selective A_2B_ agonist BAY 60-6583 (10^−6^ mol/L) did not show significant changes in superoxide production (Fig. 5F–H). Preincubation of the coronary artery with Nox inhibitor gp91 ds-tat (10^−6^ mol/L) and ERK 1/2 inhibitor PD98059 (10^−5^ mol/L) prevented adenosine and CGS-21680-mediated superoxide increase (Fig. 5G and H). These data further suggest that Nox is the source of superoxide production induced by adenosine in mouse coronary artery smooth muscle and endothelial cells. The interaction between adenosine and ROS/Nox possibly involves ERK 1/2 phosphorylation via the A_2A_AR.

## Discussion

This study shows a link between ROS generated through Nox, mainly Nox2, and its involvement in adenosine-induced increase in CF in isolated mouse hearts. Our data indicate that adenosine enhances Nox activation not by changes in protein expression of Nox isoforms but via increased phosphorylation of the p47-phox subunit. Furthermore, this study shows ERK 1/2 phosphorylation to be a possible signaling mechanism through which adenosine mediates p47-phox phosphorylation. This signaling pathway is possibly through A_2A_AR-mediated CF effects induced by adenosine.

We used adenosine as the endogenous nonselective AR agonist in this study. Adenosine plays an important role in the control of CF under different metabolic conditions (Berne 1963). As expected, adenosine induced a large increase in CF in our isolated mouse heart model, which is well documented from studies by our lab (Morrison et al. 2002; Talukder et al. 2003; Sharifi Sanjani et al. 2011; Sharifi-Sanjani et al. 2013) and others (Flood and Headrick 2001; Sato et al. 2005).

Since both ROS and adenosine are released in conditions of stress such as ischemia–reperfusion and hypoxia probably to aid restoration of CF and oxygen supply to heart, we hypothesized a possible link between adenosine-mediated effects and ROS generation. We have shown previously that A_3_AR activation induces contraction of the mouse aorta which is dependent on ROS generation, possibly through Nox2 (El-Awady et al. 2011). Several other studies have shown that inhibition or absence of Nox leads to prevention of adenosine responses in renal arterioles (Carlstrom et al. 2009), and cerebral arteries (Gebremedhin et al. 2010), confirming an important link between ROS and adenosine.

In this work, we show that Nox inhibitors apocynin and gp91 ds-tat significantly attenuate the adenosine-mediated increase in CF in WT isolated hearts, suggesting that CF increase by adenosine involves Nox activation. The gp91 ds-tat is a specific inhibitor for Nox2 (Rey et al. 2001), suggesting that adenosine mainly activates Nox2. Moreover, the SOD and catalase-mimicking agent EUK134 was able to reduce the adenosine-mediated response in a way similar to Nox inhibitors, confirming the involvement of ROS in adenosine-induced increase in CF regulation. This effect of Nox inhibition at higher concentrations of adenosine (micomolar) can occur during different physiological (such as exercise) and pathological (such as ischemia–perfusion) conditions (Berne 1963; Driver et al. 1995).

To rule out if either A_1_ or A_3_ AR is involved in a relationship between adenosine and Nox in the heart, we used A_1_ and A_3_ AR knockout mice. We used knockout animals as this approach eliminates questions on selective agonist specificity and effectiveness. In addition, selective agonists tend to lose their selectivity at higher doses. Furthermore, we used A_1_/A_3_DKO mice to avoid any compensation or upregulation of either A_1_ or A_3_ ARs, as observed in our previous study on A_2A_ and A_2B_ ARs (Teng et al. 2008; Sharifi Sanjani et al. 2011). Our results show that inhibition of Nox by apocynin or gp91 ds-tat decreased adenosine-induced CF in all three KOs similar to WT. This suggests that neither A_1_ nor A_3_ ARs are involved, but possibly A_2A_ and/or A_2B_ ARs. Future studies in our lab using A_2A_ and A_2B_ AR knockout animals would throw more light on this hypothesis.

To further confirm the relationship between adenosine and Nox, we examined how adenosine affects the protein expression of different Nox isoforms (1, 2, and 4). Our previous work in mouse aorta showed that Nox2 protein expression can be increased with A_3_AR stimulation (El-Awady et al. 2011). However, the present data suggest that adenosine induces no changes in the different Nox isoforms protein expression in isolated hearts. Because p47-phox phosphorylation plays a major role in Nox activation and regulation (Dang et al. 2006; Thakur et al. 2010), we tested p47-phox phosphorylation in our model. Adenosine treatment in isolated heart was associated with increased phosphorylation of p47-phox subunit, confirming that Nox can be activated by adenosine in isolated hearts.

To further elucidate upstream mechanisms involving p47-phox and Nox activation, we considered ERK 1/2 as a possible upstream candidate. ERK 1/2 has been shown to be activated by adenosine in newborn rat cardiomyocytes (Germack and Dickenson 2004), mouse coronary artery smooth muscle cells (CASMCs) (Shen et al. 2005; Ansari et al. 2009), and aorta (Ponnoth et al. 2012). In addition, ERK has been shown to be involved in phosphorylation of p47-phox (El Benna et al. 1996). Our data suggest that adenosine can stimulate phosphorylation of ERK 1/2 in isolated hearts. Hence, ERK 1/2 may represent an important pathway through which adenosine can activate Nox.

Vascular Nox are activated within minutes of stimulation (Seshiah et al. 2002), producing different species of ROS, mainly superoxide anion. This superoxide can enhance vasoconstriction by rapidly converting nitric oxide (NO) to the much less active vasodilator peroxynitrite (Koppenol et al. 1992). To measure the intracellular superoxide production induced by adenosine, we used the DHE fluorescence dye in mouse coronary arteries. Our results show that adenosine induces superoxide production in both mouse CASMCs and CAECs. In addition, the selective A_2A_AR agonist CGS-21680 induced a similar response as adenosine in mouse coronary artery whereas the selective A_2B_AR agonist BAY 60-6583 did not increase superoxide levels. Previous work on rat cerebral arteries (Gebremedhin et al. 2010) has shown that both A_2A_ and A_2B_ ARs are involved in adenosine-induced ROS production and vasodilation. Our results reveal that there is a subtype selectivity of A_2A_ARs involvement in ROS production in mouse coronary artery. This effect was attenuated by gp91 ds-tat, confirming that adenosine stimulates superoxide production in coronary artery (CASMCs and CAECs) through activation of Nox. Moreover, ERK 1/2 involvement was also confirmed in this AR/Nox/ROS pathway because its inhibition by PD98059 abolished the superoxide production by adenosine and its analogues.

As adenosine increased ROS in mouse coronary artery by 20%, therefore ROS is responsible for a part of adenosine responses. It is likely that there may be other signaling pathways that may contribute to this effect. Additionally, further in vivo studies will be required to examine CF responses to adenosine and contribution of myocyte-derived ROS to the adenosine-stimulated increase in CF.

A better understanding of the relationship between adenosine and ROS generation, possibly through Nox, may result in potential therapeutic targets in cardiovascular pathophysiological situations involving higher oxidative stress.

In conclusion, adenosine-induced increase in CF in isolated mouse heart involves ROS generation from Nox2. Adenosine enhances ERK 1/2 phosphorylation leading to phosphorylation and activation of p47-phox subunit with subsequent activation of Nox and release of superoxide. This relationship between adenosine and ROS/Nox involves neither A_1_ nor A_3_ ARs, but is mediated possibly through A_2A_ARs in mice. This study, for the first time, employed freshly isolated mouse coronary arteries staining for superoxide measurement. Furthermore, the study decodes the link between adenosine and ROS-mediated coronary flow responses to be mediated via A_2A_ARs.
